# Supplementary material for: Rad27/FEN1 prevents accumulation of Okazaki fragments and ribosomal DNA copy number changes
Source: FEBS Lett. 2025 Oct 27;599(24):3669–84. doi: 10.1002/1873-3468.70193 (PMC12720220; doi:10.1002/1873-3468.70193)
Supplement: Supplementary file 1 — Table S1. Saccharomyces cerevisiae strains used in this study. [file FEB2-599-3669-s001.pdf]

Supplementary Table S1. *Saccharomyces cerevisiae* strains used in this study

Name	Genotype
MSY275	*MATa*
MSY360	*MATa, NatNT2-GALL-FOB1, bar1::LEU2*
MSY409	*MATa, fob1::LEU2*
MSY937	*MATa, NatNT2-GALL-FOB1, bar1::LEU2, sir2Δ::hphMX, hmlΔ::kanMX*
MSY1645	*MATa, sir2Δ::hphMX*
MSY1648	*MATa, rad27Δ::kanMX*
MSY1651	*MATa, sir2Δ::hphMX, rad27Δ::kanMX*
MSY1654	*MATa, fob1::LEU2, sir2Δ::hphMX*
MSY1657	*MATa, fob1::LEU2, rad27Δ::kanMX*
MSY1660	*MATa, fob1::LEU2, sir2Δ::hphMX, rad27Δ::kanMX*
MSY1636	*MATa, din7Δ::kanMX*
MSY1638	*MATa, yen1Δ::kanMX*
MSY1640	*MATa, exo1Δ::kanMX*
MSY1665	*MATa, RAD27::rad27-G240D::LEU2*
MSY1674	*MATa, RAD27::LEU2*
MSY1680	*MATa, RAD27::rad27-324::LEU2*
MSY1684	*MATa, RAD27::rad27-G67S::LEU2*
MSY1687	*MATa, RAD27::rad27-E158D::LEU2*
MSY1790	*MATa, NatNT2-GALL-FOB1, bar1::LEU2, rad27Δ::kanMX*
MSY1898	*MATa, rad2Δ::kanMX*
ZYY236	*MATa/α, est2Δ::URA3/EST2*, *cdc9-1::kanMX/CDC9*

All strains are derivatives of W303, which is *ade2-1, ura3-1, his3-11, 15, trp1-1, leu2-3, 112, can1-100*, and *RAD5*.
